# Gastrointestinal Alpha Heavy Chain Disease With Persistent *Campylobacter Jejuni* Colonization and Refractory Giardiasis

**DOI:** 10.14309/crj.0000000000001467

**Published:** 2024-08-22

**Authors:** Wei Tang, Zilan Lin, Pritika Sharma, Gabriel Heering, Brad Dworkin, Amir Steinberg, Fouzia Shakil, Beth Schorr-Lesnick

**Affiliations:** 1 Department of Internal Medicine, New York Medical College, Valhalla, NY; 2 Department of Gastroenterology and Hepatobiliary Diseases, New York Medical College, Valhalla, NY; 3 Department of Hematology and Oncology, New York Medical College, Valhalla, NY; 4 Department of Pathology, New York Medical College, Valhalla, NY

**Keywords:** heavy chain disease, extranodal marginal zone lymphoma, mucosa associated lymphoid tissue lymphoma, *Campylobacter jejuni*, *Giardia lamblia*, *Helicobacter pylori*

## Abstract

Alpha heavy chain disease (αHCD) is a rare variant of the mucosa-associated lymphoid tissue lymphoma characterized by expression of a monotypic truncated immunoglobulin α heavy chain. αHCD frequently involves the gastrointestinal (GI) tract, and its pathogenesis has been linked to clonal B-cell expansion from chronic immune stimulation by infectious agents. We report a rare case of GI αHCD with 5 concomitant pathogens identified on a GI multiplex real-time polymerase chain reaction panel, featured by persistent *Campylobacter jejuni* colonization and refractory giardiasis.

## INTRODUCTION

Extranodal marginal zone lymphoma (EMZL) of the mucosa-associated lymphoid tissue (MALT), also known as MALT lymphoma, is an indolent B-cell lymphoma that most frequently involves the gastrointestinal (GI) tract. Immunoproliferative small intestinal disease (IPSID), also known as alpha heavy chain disease (αHCD), is a variant of MALT lymphoma characterized by expression of a monotypic truncated immunoglobulin α heavy chain without an associated light chain.^1^ The pathogenesis of MALT lymphoma is linked to clonal B-cell expansion from chronic immune stimulation by infectious agents.^2^ A growing list of bacteria, viruses, and parasites have been identified as potential causative agents in the pathogenesis, where chronic antigenic exposure and inflammation synergize with genetic alterations to sustain the development of a MALT lymphoma.^3^ We report a rare case of a patient with gastric and intestinal αHCD with 5 concomitant infections identified on a stool real-time polymerase chain reaction (GI multiplex), featured by persistent *Campylobacter jejuni* colonization and refractory giardiasis despite treatment.

## CASE REPORT

**Figure 1.**
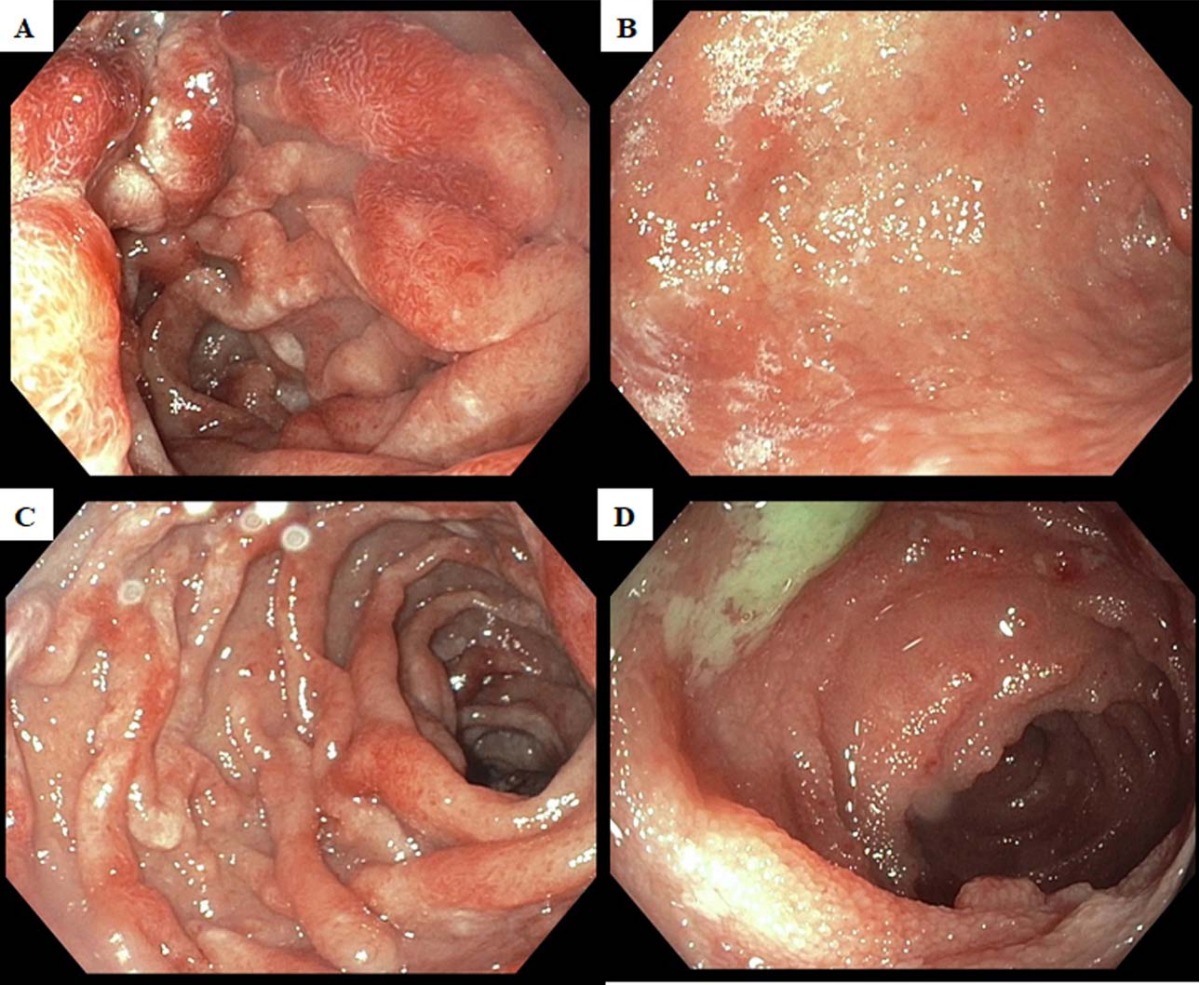
Esophagogastroduodenoscopy and colonoscopy findings. (A) Congestion, erythema, and nodularity in the anterior duodenal bulb. (B) Edema, erythema, and nodularity in the antrum and stomach body compatible with infiltrative disease. (C) Congestion, erythema, and nodularity in the second portion of the duodenum. (D) Edema, congestion, and nodularity in the terminal ileum.

**Figure 2.**
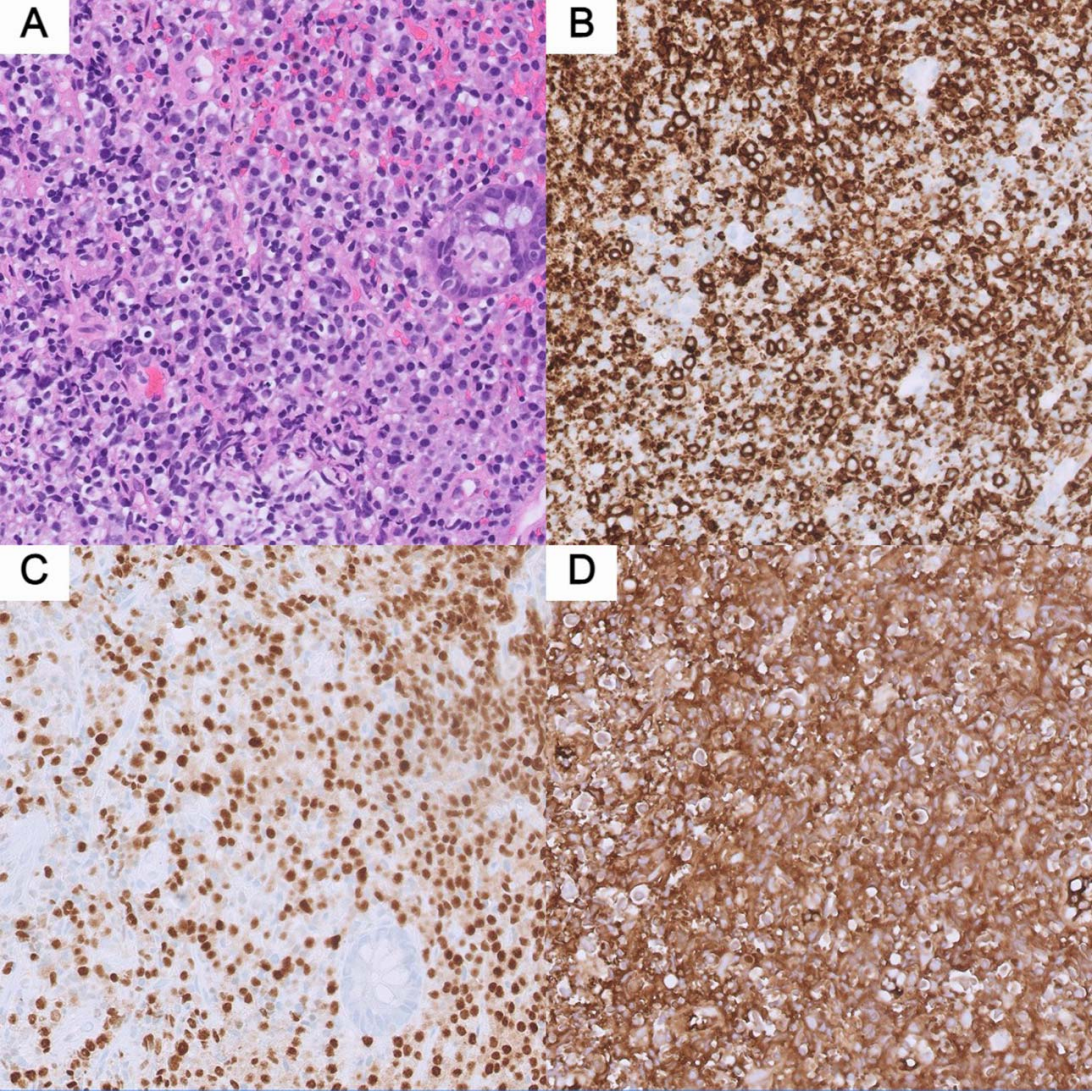
Histopathological findings of terminal ileum biopsy. (A) High magnification (20×) hematoxylin and eosin section of terminal ileum showing infiltration of the mucosa by sheets of atypical monocytoid lymphocytes. Destruction of crypts with lymphoepithelial lesion is also identified. (B) Atypical cells showing positivity for CD20. (C) Atypical cells showing positivity for multiple myeloma-1. (D) Atypical cells showing positivity for IgA.

A 34-year-old Hispanic man with 3-month history of lower abdominal pain, intermittent bloody diarrhea, and unintentional weight loss presented to our hospital for further management of suspected αHCD. Computed tomographic imaging performed at the referring institute was significant for extensive mesenteric lymphadenopathy. Endoscopic biopsy of the terminal ileum revealed extensive infiltration of the intestinal epithelium with IgA-positive, multiple myeloma-1-positive, CD20-negative plasmacytoid lymphocytes and plasma cells with aberrant absence of kappa or lambda light chain expression suggesting αHCD. We performed an esophagogastroduodenoscopy and repeat colonoscopy to confirm the diagnosis, which redemonstrated diffuse continuous edema, erythema, and nodularity in the stomach, duodenum, and entire colon (Figure 1). Biopsies of the stomach, duodenum, and terminal ileum revealed infiltration of the intestinal mucosa by sheets of atypical monocytoid lymphocytes positive for CD20, multiple myeloma-1, and IgA and destruction of crypts with lymphoepithelial lesions (Figure 2); additional stain was positive for *Helicobacter pylori*. Quantification of immunoglobulins showed isolated elevation of IgA level to 1,056 mg/dL. GI multiplex was subsequently collected for worsening diarrhea and returned strikingly positive for 5 different pathogens including *C. jejuni*, enteroaggregative *Escherichia coli*, enteropathogenic *Escherichia coli*, *Cryptosporidium* species, and *Giardia lamblia*; these results were confirmed on a repeat GI multiplex 48 hours later. Initial serological test for Epstein-Barr virus infection was negative. The patient was treated with a 3-day course of azithromycin and nitazoxanide, started on *H. pylori* eradication therapy, and initiated on bendamustine plus rituximab (BR)—a guideline-recommended first-line regimen for αHCD. His GI symptoms improved, and *H. pylori* was successfully eradicated.

**Figure 3.**
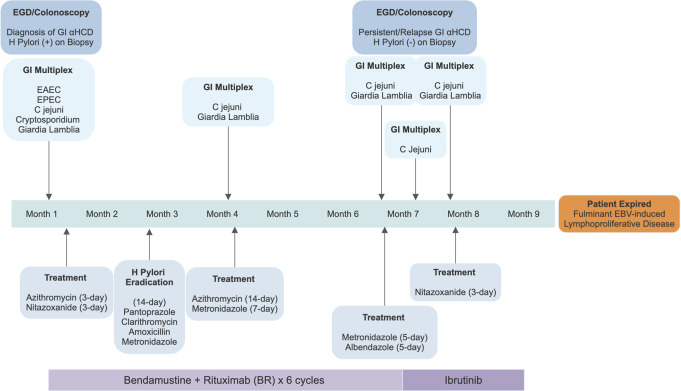
Time line of the disease course with treatments. *C.jejuni*, *Campylobacter jejuni*; EAEC, enteroaggregative *E. coli*; EBV, Epstein-Barr virus; EGD, esophagogastroduodenoscopy; EPEC, enteropathogenic *E. coli*; GI, gastrointestinal; *H. pylori*, *Helicobacter pylori*; αHCD, alpha heavy chain disease; (+), positive; (−) negative.

However, similar GI symptoms recurred 3 times during the following 9 months (Figure 3). Despite treatment with multiple antibiotics including azithromycin, metronidazole, and albendazole for prolonged courses, the patient remained positive for *C. jejuni* and giardia, suggesting persistent *C. jejuni* colonization and refractory giardiasis. In addition, repeat esophagogastroduodenoscopy/colonoscopy demonstrated similar mucosal disease, indicating no lymphoma response to 6 cycles of standard BR treatment. Ibrutinib monotherapy, a Bruton tyrosine kinase (BTK) inhibitor, was attempted as a second-line treatment after failure of response to traditional chemotherapy agents. The patient progressed to develop fulminant EBV-induced lymphoproliferative disease while on ibrutinib treatment and died of it.

## DISCUSSION

αHCD is a rare form of MALT lymphoma affecting primarily young (age 10–30 years) male individuals of Mediterranean and Middle Eastern descent. Its epidemiologic association with low socioeconomic background and poor sanitation suggests a possible infectious mechanism.^4^ A considerable portion of MALT lymphoma cases develop in the setting of chronic immune stimulation from bacterial stimuli. The prototypical example is the association of *H. pylori* infection with chronic gastritis and the development of gastric MALT lymphoma. A reminiscent link between *C. jejuni* and IPSID has been proposed based on the post hoc detection of *C. jejuni* DNA in IPSID histopathology specimens in several retrospective studies^5,6^; however, there has been no laboratory data validating the effects of *C. jejuni* on lymphoma cells or in animal models.^7^ In addition, occasional reports have also noticed associations between parasitic infection and the development of MALT lymphoma, including giardiasis. Chemoimmunotherapy with anti-CD20 monoclonal antibody such as BR has been recommended as the preferred first-line regimen for systemic therapy for EMZL.^8^ BTK inhibitors are used as second-line treatment modalities for EMZL refractory to traditional chemoimmunotherapy. However, there is paucity of data on the use of ibrutinib in αHCD. To our knowledge, this is the first case reporting the use of ibrutinib for small intestine αHCD with gastric extension after failure to BR therapy. Unfortunately, the patient died of fulminant EBV-induced lymphoproliferative disease before treatment response could be assessed. Given the historically suboptimal outcomes noted with chemoimmunotherapy in αHCD, strong consideration should be given to the incorporation of BTK inhibitors in earlier lines of treatment.^9^

Our case of GI αHCD involving both stomach and small intestine with multiple concomitant infections, including *H. pylori*, *C. jejuni*, giardia, and *E. coli*, redemonstrated the widely recognized etiopathogenic model of MALT lymphoma. To our knowledge, this is the first case of GI αHCD with 5 pathogens concomitantly identified on a single GI multiplex. We suspect the coexistence of multiple enteric pathogens, especially the presence of *Giardia lamblia*, which is suggestive of poor sanitation and contaminated drinking water, which is an established environmental risk factor of αHCD.^10^ Interestingly, this patient was also infected with *H. pylori*, a well-known microbial cause of gastric MALT; contrary to reported αHCD cases where the successful eradication of *H. pylori* was frequently followed by regression of αHCD, the patient had disease progression despite successful eradication of *H. pylori* without further recurrence.^11–13^ Therefore, we postulate that persistent *C. jejuni* infection and refractory giardiasis partially contributed to the treatment failure of BR therapy in this case, as chronic antigen exposure induced clonal B-cell proliferation and subsequent lymphoma progression. Some experts recommend attempting a trial of empiric antibiotic therapy for a minimum of six-month course with metronidazole, ampicillin, or tetracycline even in the absence of a documented enteric infection, to improve malabsorption symptoms and assess for disease responsiveness in all types of EMZL.^14^ Further studies are needed to investigate the degree of immune activation from enteric infection in the development of αHCD and identify more microorganisms with lymphomagenic capability. Patients with GI αHCD who fail the standard treatments should be thoroughly screened for enteric infections. Different diagnostic modalities should be considered, including both molecular techniques and culture-based methods. Prudent evidence is needed to validate and standardize the antimicrobial therapy in αHCD with or without enteric pathogen infections.

## DISCLOSURES

Author contributions: All named authors meet the International Committee of Medical Journal Editors (ICMJE) criteria for authorship for this article, take responsibility for the integrity of the work as a whole, and have approved this version for publication. B. Schorr-Lesnick, B. Dworkin, and A. Steinberg contributed to the concept and design of the manuscript. W. Tang, P. Sharma, Z. Lin, G. Heering, and F. Shakil all contributed to the composition and edits of the manuscript. B. Schorr-Lesnick is the article guarantor.

Financial disclosure: None to report.

Informed consent was obtained for this case report.

Prior presentation: This case was previously presented and selected as Presidential Poster Award at ACG 2023 Annual Scientific Meeting on October 20–25, 2023 in Vancouver, British Columbia, Canada.
